# Modifying Effects of the *HFE* Polymorphisms on the Association between Lead Burden and Cognitive Decline

**DOI:** 10.1289/ehp.9855

**Published:** 2007-05-10

**Authors:** Florence T. Wang, Howard Hu, Joel Schwartz, Jennifer Weuve, Avron S. Spiro, David Sparrow, Huiling Nie, Edwin K. Silverman, Scott T. Weiss, Robert O. Wright

**Affiliations:** 1 Department of Environmental Health, Harvard School of Public Health, Boston, Massachusetts, USA; 2 Department of Environmental Health Sciences, University of Michigan School of Public Health, Ann Arbor, Michigan, USA; 3 Channing Laboratory, Brigham and Women’s Hospital, Boston, Massachusetts, USA; 4 Department of Epidemiology, Harvard School of Public Health, Boston, Massachusetts, USA; 5 VA Boston Healthcare System, Boston, Massachusetts, USA; 6 Department of Epidemiology, Boston University School of Public Health, Boston, Massachusetts, USA; 7 Department of Medicine, Boston University School of Medicine, Boston, Massachusetts, USA; 8 Department of Medicine, Children’s Hospital Boston, Boston, Massachusetts, USA

**Keywords:** cognitive decline, epidemiology, *HFE*, lead, longitudinal studies, neuropsychologic tests

## Abstract

**Background:**

As iron and lead promote oxidative damage, and hemochromatosis (*HFE*) gene polymorphisms increase body iron burden, *HFE* variant alleles may modify the lead burden and cognitive decline relationship.

**Objective:**

Our goal was to assess the modifying effects of *HFE* variants on the lead burden and cognitive decline relation in older adults.

**Methods:**

We measured tibia and patella lead using K-X-ray fluorescence (1991–1999) among participants of the Normative Aging Study, a longitudinal study of community-dwelling men from greater Boston. We assessed cognitive function with the Mini-Mental State Examination (MMSE) twice (1993–1998 and 1995–2000) and genotyped participants for *HFE* polymorphisms. We estimated the adjusted mean differences in lead-associated annual cognitive decline across HFE genotype groups (*n* = 358).

**Results:**

Higher tibia lead was associated with steeper cognitive decline among participants with at least one *HFE* variant allele compared with men with only wild-type alleles (*p* interaction = 0.03), such that a 15 μg/g increase in tibia lead was associated with a 0.2 point annual decrement in MMSE score among *HFE* variant allele carriers. This difference in scores among men with at least one variant allele was comparable to the difference in baseline MMSE scores that we observed among men who were 4 years apart in age. Moreover, the deleterious association between tibia lead and cognitive decline appeared progressively worse in participants with increasingly more copies of *HFE* variant alleles (*p-*trend = 0.008). Results for patella lead were similar.

**Conclusion:**

Our findings suggest that *HFE* polymorphisms greatly enhance susceptibility to lead-related cognitive impairment in a pattern consistent with allelelic dose.

In the United States the population of persons age 65 years and older is projected to increase 2-fold to 75 million in the next 30 years, and a concomitant upsurge in the number of individuals with dementia is expected (U.S. Census Bureau 2000a, 2000b). Cognitive decline, a risk factor for dementia, may be a transition stage spanning normal cognition and onset of diseases associated with dementia (Bischkopf et al. 2002; Burns and Zaudig 2002; Pratico et al. 2002).

Lead has long been recognized as a neuro-toxicant; the associations between lead and cognitive impairment among workers in lead-related industries and poorer cognitive development in children have been well reproduced (Barth et al. 2002; Bleecker et al. 1997; Lanphear et al. 2000). The few studies that have been conducted among low-level lead-exposed older adults have generally reported inverse associations between lead burden and cognitive function (Muldoon et al. 1996; Nordberg et al. 2000; Payton et al. 1998; Weisskopf et al. 2004; Wright et al. 2003). With half-life estimates ranging from 5 to 20 years for cortical bone, and more than1 year for trabecular bone (Hu et al. 1998; Kim et al. 1997), lead levels in bone may better reflect long-term body burden of lead than blood lead, which has a half-life of approximately 30 days (Hu 2001). In addition, bone lead measures correlate well with measures of cumulative external lead levels and integrated blood lead levels, two commonly used indices of cumulative lead exposure (Bleecker et al. 1997).

Iron metabolism may play a critical role in neurodegenerative processes (Lee et al. 2006; Todorich and Connor 2004). Although iron is vital for cellular processes, plasma iron which is not bound to transferrin may be toxic. This nontransferrin-bound iron represents the portion of body iron likely to cause cellular oxidative damage, a purported mechanism in the pathogenesis of neurodegenerative diseases, and serve as a catalyst in the neuronal production of free radicals (Eaton and Qian 2002; Samson and Nelson 2000). Two variants in the hemochromatosis (*HFE)* gene, *C282Y* and *H63D*, are commonly found in the U.S.population, especially among whites, and are associated with hereditary hemochromatosis, a disease of iron overload. Several recent studies have reported an association between these *HFE* polymorphisms and neurodegenerative diseases such as Alzheimer disease (Berlin et al. 2004; Moalem et al. 2000; Sampietro et al. 2001). Furthermore, carriers of these polymorphisms who do not have clinical signs of iron overload are observed to have higher levels of nontransferrin-bound iron in addition to body iron measures higher than wild-types (Adams et al. 2005; Beutler et al. 2003; Datz et al. 1998; de Valk et al. 2000; Garry et al. 1997).

These studies have led to a growing interest in the interaction of iron and lead metabolism in the process of neurodegeneration. As *HFE* variant alleles are associated with neurodegenerative processes similar to those seen in lead toxicity, and the presence of iron enhances the oxidative effects of lead (Adonaylo and Oteiza 1999), *HFE* variant alleles may magnify the neurologic damage caused by lead. Therefore, we examined the modifying effect of the HFE alleles on the association between body lead burden and change in performance on the Mini-Mental State Examination (MMSE), a test of global cognitive function, in a cohort of older, community-dwelling men.

## Materials and Methods

Participants in the current study were drawn from the Normative Aging Study (NAS), a community-based, prospective cohort study initiated in 1963 at the Veterans Affairs (VA) Outpatient Clinic in Boston to examine factors related to healthy aging (Bell et al. 1972). The cohort consisted of 2,280 men 21–81 years of age at the time of enrollment (1963–1968) who had successfully completed a screening process to ensure participants were free of known chronic medical conditions. Most cohort members were of northern European descent. Overall, their smoking and alcohol consumption patterns were similar to men of comparable age in the U.S.population. Every 3–5 years, study participants were asked to undergo extensive evaluations including medical and physical examinations and laboratory tests. They also completed questionnaires on smoking history, diet, and other factors potentially related to aging and health. To date, the annual attrition due to all causes has been less than 1%, and more than 80% have responded to mailed questionnaires supplementing on-site examinations (Hu et al. 1996). This study has been approved by the Human Subjects Committees of the Boston VA Medical Center, the Brigham and Women’s Hospital, and the Harvard School of Public Health.

### Study population

Beginning in 1991, bone lead measurements were taken using K-X-ray fluorescence (KXRF) among active participants who gave written informed consent. In 1993, cognitive function assessments were initiated. At the time of the present study, 1,055 study participants had completed at least one cognitive assessment; whereas 540 men had two or more assessments. The first and second cognitive assessments were on average 3.2 years apart. Of the men with two cognitive measures, 420 had at least one bone lead measurement. In 2000, NAS participants (*n* = 730) were genotyped for two *HFE* polymorphisms based on archived blood samples. In all, our analyses included the 358 men with at least two cognitive assessments, complete covariate information, *HFE* genotyping data, and at least one measure of bone lead.

### Bone lead KXRF measurement

*In vivo* bone measurements were taken using a K-X-ray fluorescence (KXRF) instrument (ABIOMED, Inc., Danvers, MA) at the mid-tibia (shin bone) and the patella (knee cap bone) (Aro et al. 2000). The sites were chosen to be representative of the two predominant bone types: cortical bone (tibia) and trabecular bone (patella). These measurements had units of micrograms of lead per gram bone mineral. The bone measurement taken closest in time to the baseline cognitive assessment served as a proxy for tissue lead burden. The instrument also provides an estimate of the uncertainty for each measurement equivalent to the standard deviation of repeated measurements. Lead estimates with uncertainty values > 10 μg/g for tibia and > 15 μg/g for patella were excluded as unreliable, a standard protocol in analyses of bone lead (Hu et al. 1998). Negative estimates of bone lead concentrations may occur for lead values close to zero. As recoding the negative values to the minimum detectable limit may induce bias and reduce efficiency in the statistical analyses, KXRF-measured bone lead concentration estimates were used in the analysis without recoding (Kim et al. 1997).

### HFE genotyping

We genotyped participants for both the *C282Y* and *H63D* polymorphisms of the *HFE* gene (GenBank accession no. Z92910; http://www.ncbi.nlm.nih.gov/sites/entrez) using archived blood. Puregene DNA isolation kits (Gentra Systems, Inc., Minneapolis, MN) were used to extract the DNA from the blood sample. The *H63D* polymorphism was genotyped by polymerase chain reaction (PCR) followed by restriction fragment length polymorphism (RFLP) analysis as previously described (Cardoso et al. 1998; Wright et al. 2004). Similarly, the *C282Y* polymorphism was genotyped by separate PCR and RFLP procedures (Cardoso et al. 1998; Feder et al. 1996).

As a quality control measure, 10% of samples were randomly selected and run in duplicate. Genotypes were also determined on control blood known to be from persons homozygous for the wild-type genotype and heterozygous and homozygous for each *HFE* variant genotype. The full data set was anonymized after genotyping to protect our study members and to conform to current Institutional Review Board policies.

### Assessment of cognitive function

One of the tests included in the cognitive assessment battery was the MMSE, a global examination of cognitive function that assesses orientation, immediate and short-term recall, verbal and written skills, and attention and ability to follow commands (Crum et al. 1993; Folstein et al. 1975). The test is commonly used in epidemiologic studies to evaluate cognitive status (Farmer et al. 1995; Izaks et al. 1995; Knopman et al. 2003). Scores range from 0 to 30, with higher score denoting better cognitive performance, although in our analysis the highest possible score was 29 because of deletion of the question “What county are we in?” from our tally. Other studies have reported that most Massachusetts residents do not know in which county they reside as counties in Massachusetts do not have strong governmental function (Tombaugh and McIntyre 1992).

### Statistical analysis

Because of small sample sizes in some strata of *HFE* genotypes, we classified *HFE* genotypes in two different manners: binary [wild-type (having only *HFE* wild-type alleles), any *HFE* variant allele]; and dose (wild-type, one *HFE* variant allele, two *HFE* variant alleles). Our measure of change in cognition was the average annual rate of decline in MMSE score, defined as (MMSE score at second visit – MMSE score at baseline visit)/(years between assessments), for each participant.

We analyzed lead levels in tibia and patella separately. To assess effect modification, we fitted multiple linear regression models of average annual rate of decline in MMSE score, in which we included a term for the lead bio-marker, indicator variables for the *HFE* genotype classification, and cross-product terms between *HFE* genotype and lead biomarker, along with terms for age, years of education, smoking status (current, never, past), pack-years smoked, nondrinker, alcohol consumption (grams/day), English as first language (yes, no), computer experience (yes, no) and diabetes (diagnosis or fasting glucose > 126 mg/dL). Values of covariates used in the analyses were those reported at the baseline MMSE assessment. Stroke and Alzheimer disease predict MMSE score, but as so few men in our study population had such conditions, these conditions were not considered in our analyses. We assessed the linearity of the association between lead and annual rate of cognitive decline within class of *HFE* genotype by fitting a penalized spline for the lead biomarker and adjusting for covariates using the generalized additive models function in R software (http://www.r-project.org/). A penalized spline is a technique for flexibly modeling dose–response by dividing the range of exposure into intervals, and fitting a separate cubic polynomial within each interval. A penalty term is added to the log likelihood that is proportional to how “wiggly” the resulting dose–response curve is, which prevents excessive nonlinearity (Wood and Augustin 2002). The optimal degree of smoothing was determined by the generalized cross-validation criterion, which is, in practice, an approximation of Akaike’s information criterion (Wood and Augustin 2002).

To assess whether participants with *C282Y* and *H63D* alleles have different lead-associated cognitive changes, we also conducted exploratory analyses to evaluate the association of lead on cognitive decline by *HFE* genotype groups (e.g., wild-type, *H63D* homozygotes, *C282Y* heterozygotes). Finally, to assess the robustness of our results, we first restricted our analyses to white participants, then repeated the analyses after removing outliers identified by the generalized extreme studentized deviation (ESD) method (Rosner 1983). All statistical analyses were conducted using SAS (version 8.2; SAS Inc., Cary, NC) and R version 2.1.1. We used partial F-tests and likelihood ratio tests for statistical hypothesis testing. The *p*-value of significance was < 0.05.

## Results

Median concentrations of bone lead in our study population were 19 and 23 μg/g for tibia and patella, respectively. Participants who were younger, more educated, native English speakers, or who had computer experience on average had lower bone lead levels (Table 1).Bone lead concentrations were slightly higher among men with lower baseline cognitive scores. Thirty-six percent of men had at least one *HFE* variant allele.Both genotype distributions conformed to Hardy-Weinberg expected frequencies (*C282Y*: χ^2^ =0.53, *p* = 0.47; *H63D*:χ^2^ =0.18, *p* = 0.67). Nine participants in our population were heterozygous for both *C282Y* and *H63D* polymorphisms (known as compound heterozygotes). Although bone lead levels appeared higher in the four participants who were *C282Y* homozygotes, these measures were not significantly different than the bone lead levels among wild-types. Characteristics of men with only *HFE* wild-type alleles were similar to those of variant allele carriers with the exception of report of computer experience (Table 2). Compared with men in the larger NAS cohort who had only one MMSE score, men in our study population had similar average baseline MMSE scores and only slightly lower mean bone lead levels (data not shown). The distribution of *HFE* genotypes was also similar in these two groups. Men with and without bone lead measures had similar baseline characteristics, distribution of *HFE* genotypes, and MMSE scores (data not shown).

**Figure 1:**
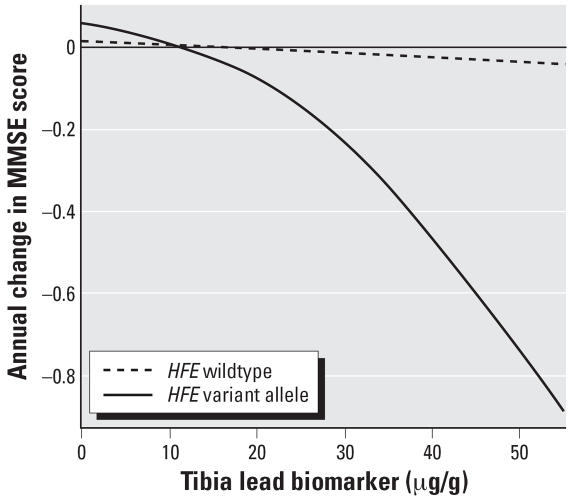
Exploration of nonlinear association of tibia lead concentration with annual rate of cognitive decline, by class of *HFE* genotype. The lines indicate curvilinear trends estimated from the penalized spline method. Among *HFE* wild-types, the optimal degree of smoothing was 1, meaning that the association between tibia lead and annual cognitive decline was nearly linear, but among variant allele carriers, the association tended to deviate from linearity (*p* = 0.08), with an optimal 1.68 degree of smoothing. The model was adjusted for age, years of education, nonsmoker, former smoker, pack-years, nondrinker, alcohol consumption, English as first language, computer experience, and diabetes.

The average annual rate of decline in MMSE scores was modestly though not significantly worse among *HFE* variant allele carriers than among men with only *HFE* wild-type alleles {difference in annual rate of change: –1.77 points/year [95% confidence interval (CI), –3.88 to 0.35]}, results not shown). Higher tibia lead was associated with a steeper decline in MMSE scores among participants with at least one *HFE* variant allele compared with wild-types (*p* = 0.03; Table 3, model 1). Among participants with any *HFE* variant allele, an interquartile range (IQR) increment in tibia lead (15 μg/g) was associated with a 0.22-point annual decrement in MMSE score (95% CI, –0.39 to –0.05). This annual change in MMSE score among variant allele carriers was approximately equivalent to the difference in baseline MMSE scores between men in our study population who were 4 years apart in age. In contrast, although tibia lead level was associated with decline in MMSE score among wild-types as well, this association was smaller in magnitude and not statistically significant. We used covariate-adjusted penalized splines to explore the linearity of the association between tibia lead level and change in MMSE score by *HFE* genotype, as shown in Figure 1. Even though the association of lead burden with cognitive decline among men with only *HFE* wild-type alleles appeared linear (optimal degree of smoothing: 1, *p* = 0.72), the association appeared curvilinear, specifically, steeper with greater lead burden in the variant allele carriers. The test for deviation from linearity for the tibia lead–cognitive change association among the variant allele carriers was nearly significant (optimal degree of smoothing: 1.68, *p* = 0.08), suggesting that, among variant allele carriers, a unit increase in lead burden may be associated with disproportionately greater cognitive decline at high lead burden than at low lead burden.

Interestingly, the detrimental association between tibia lead and decline in MMSE was progressively larger with increasing number of *HFE* variant alleles: each IQR increment in tibia lead was associated with a –0.02-point/year change in MMSE score among wild-types, –0.14-point/year change among men with one *HFE* variant allele, and –0.63 point/year change among men with two *HFE* variant alleles (*p <* 0.01, Table 3, model 2). The pronounced difference in lead-associated cognitive decline between men with two variant alleles and men with only wild-type alleles was significant (*p* < 0.01, Table 3, model 2). This difference in change in MMSE scores among participants with two *HFE* variant alleles was comparable to the difference in baseline MMSE scores that we observed among men who were 11 years apart in age.

We also examined the modification of the lead association with annual change in MMSE score by *HFE* genotype groups. The magnitudes of effect modification among *H63D* heterozygotes and *C282Y* heterozygotes were very similar to the magnitude of effect modification among one variant allele carriers. Similarly, the results for *H63D* homozygotes and *C282Y* homozygotes were similar to the results for two variant allele carriers (results not shown).

Overall, the associations pertaining to patella lead were similar to although smaller in magnitude than those pertaining to tibia lead (results not shown). Results from analyses in which we excluded extreme values of bone lead were also similar, as were results in analyses in which we restricted the study population to whites (results not shown).

## Discussion

In our population of older men, the deleterious association between long-term lead burden and rate of decline in cognitive function was significantly worse among *HFE* variant allele carriers than among wild-types. Furthermore, the detrimental association of lead with cognitive decline was magnified among participants with a greater number of either variant alleles (*H63D* or *C282Y* ); the largest drop in MMSE scores associated with lead burden was observed in men carrying two variant alleles. Of note, the magnitude of effect modification was linked to number and not to type of *HFE* variant alleles. Our study is the first to provide evidence that the neurodegenerative effects of these variants during aging may be in part due to genetic susceptibility to non-iron metals such as lead.

Several studies have addressed cognitive function among adults who experienced chronic low-level lead exposure (Muldoon et al. 1996; Weisskopf et al. 2004; Wright et al. 2003). Among the studies that used bone lead measures, tibia lead was more strongly associated with cognitive decline than were patella and blood leads (Payton et al. 1998; Weisskopf et al. 2004), suggesting that lead in tibia serves as a superior proxy for effective lead dose in the brain. As lead in the tibia has a substantially longer half-life than lead in the patella or blood (Hu 1998), these results indicate that long-term, chronic lead exposure may be more predictive of cognitive changes. Interestingly, the strongest interaction between lead and *HFE* variant alleles in our study was also observed with tibia lead measures.

We are not aware of any other study that has evaluated the modifying effect of the *HFE* genotype on the association between lead and change in cognitive function. The HFE protein appears to regulate metal transport across cell membranes (Chung and Wessling-Resnick 2003), although its role in transporting iron and non-iron divalent metals across the blood brain barrier is unknown. Two common polymorphisms in the *HFE* gene, the *C282Y* and the *H63D*, have been implicated in hereditary hemochromatosis, a disease associated with excess iron absorption (Feder et al. 1996; Hanson et al. 2001). In addition, persons who carry the *HFE* variant alleles but who lack clinical signs of hemochromatosis disease are reported to have significantly higher values of serum iron, transferrin saturation, and non-transferrin-bound iron than individuals with only *HFE* wild-type alleles (Beutler et al. 2003; Datz et al. 1998; Garry et al. 1997; Moura et al. 1998). *HFE* polymorphisms have also been reported to affect lead uptake (Bannon et al. 2003; Barton et al. 1994; Onalaja and Claudio 2000; Wright et al. 2004). In our analysis, men who were homozygous for *C282Y* appeared to have higher bone lead burden than men with other *HFE* genotypes, although we were not able to detect a statistically significant difference with our small sample size of *C282Y* homozygotes. This subject matter merits further examination in larger study populations.

Although the relationship between *HFE* alleles and neurodegenerative diseases is not fully established, several recent studies have found positive associations between *HFE* variant alleles and Alzheimer disease. Researchers have reported the *C282Y* and *H63D* polymorphisms to be significantly more prevalent in subjects with Alzheimer disease compared with controls, and that persons with Alzheimer disease and *HFE* variant alleles were on average 6 years younger at the time of diagnosis than individuals with Alzheimer disease but with only the wild-type alleles (Moalem et al. 2000; Pulliam et al. 2003; Sampietro et al. 2001). Another study also observed earlier age of onset of Alzheimer disease but only among *H63D* homozygotes (Berlin et al. 2004). In contrast, others have found no association between *HFE* alleles and Alzheimer disease (Berlin et al. 2004; Guerreiro et al. 2006). The discrepancies across studies may reflect that *HFE* polymorphisms do not independently impart cognitive risk but instead enhance the neurotoxicity of agents such as lead. Interestingly, although the *C282Y* and *H63D* functional polymorphisms may differentially alter iron and divalent metal metabolism (Bomford 2002; Lyon and Frank 2001; Townsend and Drakesmith 2002), we observed these two polymorphisms to have comparable magnitudes of effect modification on the relation between lead and cognitive decline.

It is not known how the *HFE* variant alleles may accelerate cognitive decline in the presence of lead. Lead and free iron are independently capable of promoting oxidative damage, a purported mechanism in the pathogenesis of neurodegenerative disease (Jellinger 1999; Jenner 1993; Samson and Nelson 2000; Winterbourn 1995). *In vitro* studies have suggested synergistic oxidative effects between lead and iron; lead appears to increase lipid oxidation in the presence of iron (Adonaylo and Oteiza 1999). Therefore, our findings may reflect the complex relationship between iron and lead metabolism where the metals interact to further increase damage and, consequently, cognitive decline. Unfortunately, we were not able to measure body iron status or magnitude of oxidative stress for our study population.

Our current findings are an interesting juxtaposition to our previous report in which *HFE* variant alleles were associated with lower levels of internal lead dose biomarkers in the same cohort (Wright et al. 2004). If the *HFE* variant alleles affect cognition predominantly by lowering lead accumulation in the body tissues, one might expect the variant alleles to be associated with better cognitive performance. Instead, we found a suggestive association between *HFE* variant alleles and poorer cognitive function that is consistent with the prior reports linking HFE variant alleles to neurode-generation. In addition, because the *HFE* variant alleles are not known to affect the relation of lead levels in tibia to lead levels in the brain, one would anticipate similar magnitudes of change in MMSE scores per unit increase in body lead burden among wild-types compared with variant allele carriers. Our data suggest quite the opposite, indicating that *HFE* variant alleles augment the toxicity of lead that is absorbed.

There were several limitations to this study. Although the associations observed were significant, it is essential to attempt to reproduce such associations in larger populations. The mean interval between the two cognitive tests was 3.2 years. With a longer interval between testing or with additional MMSE assessments, we may be able to better describe the relationship between lead and cognitive changes by *HFE* genotype.

As with any aging cohort, there was a potential for selection bias mainly because of differential attrition and survival. We were somewhat reassured, as the lead biomarker levels and first MMSE scores in persons who had completed only baseline cognitive assessment were similar to those found in our study population. The frequencies of *HFE* genotypes were also similar in the two groups. Moreover, baseline characteristics and MMSE scores did not differ in men with and without bone lead measures.

The potential for misclassification should be considered. Although there may have been some measurement error in our bone lead data, such errors would most likely be nondifferential, and thus, bias our association estimates toward the null. It is possible that the modifying effects observed were caused by other polymorphisms in the *HFE* gene or polymorphisms in a proximal gene that is in tight linkage disequilibrium with these *HFE* polymorphisms, although we believe this is unlikely, as we did not find other genes known to regulate iron metabolism in this genomic region.

As in any observational study, confounding is a concern. We accounted for known strong predictors of cognitive function. As only one participant had suffered a stroke and none had been diagnosed with Alzheimer disease, we did not adjust for these factors in our final model. Of the men in our study population, 99% were white, making population stratification an unlikely confounder. Overall, crude and adjusted comparisons of MMSE change were similar, suggesting that strong confounding from an unmeasured source is unlikely.

The MMSE is widely used to screen for dementia and has frequently been used to assess cognitive status and track longitudinal changes in cognitive function (Farmer et al. 1995; Izaks et al. 1995; Knopman et al. 2003). However, it is a relatively easy test for which a learning effect has been reported (Jacqmin-Gadda et al. 1997). A low degree of variability in MMSE scores among more highly educated persons is often observed (Crum et al. 1993). Therefore, the MMSE may have had low sensitivity in detecting cognitive impairment in our participants. Despite these limitations, the MMSE is among the most extensively characterized and most widely used tests of cognitive status for older adults, and performs with a reasonable degree of reproducibility and validity. Past studies have found high correlations between MMSE scores and scores on other well-described cognitive tests, such as the Blessed Information-Memory-Concentration test and reasonable sensitivity and specificity in delineating individuals with and without dementia (Crum et al. 1993; Stuss et al. 1996; Tombaugh and McIntyre 1992). Finally, persons with mild cognitive impairment are found to have significantly worse MMSE scores than cognitively normal individuals (Bennett et al. 2002).

Although we were not yet able to assess development of dementia in our population, cognitive decline is a strong predictor of subsequent dementia. Cognitively impaired individuals have a 10–15% annual risk of developing dementia compared with a 1–2% annual risk among healthy controls (Bischkopf et al. 2002; Knopman et al. 2003; Morris et al. 2001). Cognitive impairment has also been associated with a 3.1- to 5-fold increase in risk of developing Alzheimer disease, the most common cause of age-related dementia (Tuokko et al. 2003).

Our findings suggest that cumulative lead exposure may be particularly detrimental to the cognitive well-being of *HFE* variant allele carriers as they age. Given the high prevalence of variant allele carriers in North American and European populations and the long retention of lead in the body, our results indicate that lead-related cognitive impairment experienced by a large subset of older adults is likely more substantial than currently recognized. Although these early cognitive changes may have slight consequences on many affected individuals, these small decrements pose a major public health concern, translating into a much larger proportion of older individuals who are considered clinically impaired, a societal burden that is projected to grow substantially, given that older persons make up one of the fastest growing segments of our population (U.S. Census Bureau 2000a, 2000b). Our findings provide insight on the mechanisms and pathways of cognitive decline. As there is currently no cure for dementia, elucidating the biological mechanisms may facilitate the development of preventive measures and treatments to hinder the rate of cognitive decline. As long-term chronic lead exposure appears to be most detrimental to cognitive health in the later years, our findings stress the continued importance of public health interventions aimed at reducing occupational and environmental exposure to lead in younger populations including lead abatement efforts and lead exposure prevention programs.

In summary, we have found the *HFE* polymorphisms to significantly modify the association between lead burden and the rate of cognitive decline. Persons with more copies of *HFE* variant alleles experienced greater cognitive decline per unit increase in bone lead biomarker level.

## Figures and Tables

**Table 1:** Baseline characteristics of study participants (*n* = 358) by bone lead measures [median μg/g (IQR)].

	Tibia lead	Patella lead
Age (years)		
< 65	15 (9–20)	18 (13–27.5)
65–70	20 (14–28)	25 (17–34)
≥71	25 (18–35)	28 (17–52)
Education		
Never finished high school	30 (14–36)	33.5 (23–46)
High school graduate	21 (14–29)	27 (16–44)
Some college	19 (13–28)	25 (15–37)
College graduate	17 (11–22)	19 (13–29)
Smoking status		
Never	17 (11–27)	21.5 (13–33)
Former	20 (14–29)	24 (16–36)
Current	19 (13.5–23.5)	27 (15–34)
Alcohol consumption		
Yes	19 (13–26)	23 (15–35)
No	20 (12.5–30)	24 (17–36)
History of diabetes^a^		
Yes	23 (14–35)	27 (17–38)
No	19 (13–27)	23 (15–35)
English as first language		
Yes	19 (12–27)	22.5 (15–34)
No	24 (15–30)	27 (17–39)
Computer experience		
Yes	15 (10–22)	20 (13.5–30)
No	21 (14–30)	26 (16–39)
Baseline MMSE score^b^		
< 26	20.5 (13–30)	26.5 (15–39)
26–27	21 (13.5–27.5)	24 (15–38)
≥28	16 (11–22.5)	21 (15–31)
*HFE* genotype		
Wild-type (*n* = 228)	19 (13–29)	24 (15–37)
One or more HFE variant alleles	19.5 (12–26)	22 (14–34)
*H63D* heterozygotes (*n* = 69)	20.5 (11.5–25.5)	22 (14–33)
*H63D* homozygotes (*n* = 8)	17 (12.5–27)	23.5 (13–34)
*C282Y* heterozygotes (*n* = 40)	17 (13–21)	22 (14–34)
*C282Y* homozygotes (*n* = 4)	32.5 (21–39)	39 (16–61.5)
Compound heterozygotes (*n* = 9) (*C282Y* and *H63D* alleles)	15 (11–20)	21 (12–27)

IQR, interquartile range.

a History of diabetes defined as having reported diagnosis of diabetes or having fasting glucose above 126 mg/dL.

b Highest possible MMSE score in our analysis was 29 because of deletion of the question “What county are we in?”

**Table 2:** Baseline characteristics of participants by *HFE* genotype (*n* = 358).

	*HFE* wild-type (*n* = 228)	*HFE* variant allele (*n* = 130)
Age [median years (IQR)]	67.2 (62.6–71.8)	67.7 [63.7–71.3]
Education [*n* (%)]		
Never finished high school	19 (8.3)	9 (6.9)
High school graduate	64 (28.1)	36 (27.7)
Some college	64 (28.1)	33 (25.4)
College graduate	81 (35.5)	52 (40.0)
Smoking status [*n* (%)]^a^		
Never	76 (33.3)	46 (35.4)
Former	138 (60.5)	81 (62.3)
Current	14 (6.1)	3 (2.3)
Alcohol consumption [median g/day (IQR)]	5.8 [0.4–18.7)	6.0 [0–16.7)
History of diabetes^b^ [*n* (%)]	24 (10.5)	13 (10.0)
English as first language [*n* (%)]	199 (87.3)	118 (90.8)
Computer experience [*n* (%)]	87 (38.2)	65 (50.0)
Patella lead [median μg/g (IQR)]	24 (15, 37)	22 (14, 34)
Tibia lead [median μg/g (IQR)]	19.0 (13, 29)	19.5 (12, 26)
Baseline MMSE score [median (IQR)]^c^	27 (25, 28)	27 (26, 28)

IQR, interquartile range.

a Percentages may not add up to 100 because of rounding.

b History of diabetes defined as having reported diagnosis of diabetes or having fasting glucose above 126 mg/dL.

c Highest possible MMSE score in our analysis was 29 because of deletion of the question “What county are we in?”

**Table 3:** Association with an interquartile (15 μg/g) increase in tibia lead biomarkers on change in MMSE score by class of *HFE* genotype.

Model/class of *HFE* genotype	Unadjusted mean difference in annual rate of change in MMSE (95% CI)	Adjusted^a^ mean difference in annual rate of change of MMSE (95% CI)	*p*-Value interaction	*p*-Value trend
Model 1: binary			0.03^b^	NA
Wild-type	–0.02 (–0.10 to 0.05)	–0.02 (–0.10 to 0.07)		
Any *HFE* variant allele	–0.23 (–0.40 to –0.07)	–0.22 (–0.39 to –0.05)		
Model 2: dose			< 0.01^c^	< 0.01
Wild-type	–0.02 (–0.10 to 0.05)	–0.02 (–0.10 to 0.07)		
One *HFE* variant allele	–0.15 (–0.33 to 0.03)	–0.14 (–0.33 to 0.04)		
Two *HFE* variant alleles	–0.62 (–1.03 to –0.22)	–0.63 (–1.04 to –0.21)		

NA, not applicable.

a Adjusted for age, years of education, nonsmoker, former smoker, pack-years, nondrinker, alcohol consumption, English as first language, computer experience, and diabetes.

b *p*-Value for tibia lead and any HFE variant allele interaction.

c *p*-Value for tibia lead and two *HFE* variant alleles interaction.
